# Initial treatment is associated with improved survival and end-of-life outcomes for patients with pancreatic cancer: a cohort study

**DOI:** 10.1186/s12885-022-10342-8

**Published:** 2022-12-14

**Authors:** Rishad Khan, Misbah Salim, Peter Tanuseputro, Amy T. Hsu, Natalie Coburn, Julie Hallet, Robert Talarico, Paul D. James

**Affiliations:** 1 Department of Medicine, University of Toronto, Toronto, Ontario Canada; 2 Division of Gastroenterology, University Health Network, University of Toronto, Room 9N-981, 200 Elizabeth Street, Toronto, ON M5G 2C4 Canada; 3 ICES, Toronto, Ontario Canada; 4 Department of Medicine, University of Ottawa, Ottawa, Ontario Canada; 5 Ottawa Hospital Research Institute, Ottawa, Ontario Canada; 6 Bruyère Research Institute, Ottawa, Ontario Canada; 7 Department of Family Medicine, University of Ottawa, Ottawa, Ontario Canada; 8 Division of General Surgery, University of Toronto, Toronto, Ontario Canada; 9 Odette Cancer Centre, Sunnybrook Health Sciences Centre, Toronto, Ontario Canada

**Keywords:** Pancreatic cancer, end-of-life care, population-based research

## Abstract

**Background:**

We describe the association between initial treatment and end-of-life (EOL) outcomes among patients with pancreatic ductal adenocarcinoma (PDAC).

**Methods:**

This population-based cohort study included patients with PDAC who died from April 2010–December 2017 in Ontario, Canada using administrative databases. We used multivariable models to explore the association between index cancer treatment (*no cancer-directed therapy, radiation, chemotherapy, surgery alone, and surgery and chemotherapy*), and primary (mortality, healthcare encounters and palliative care) and secondary outcomes (location of death, hospitalizations, and receipt of chemotherapy within the last 30 days of life).

**Results:**

In our cohort (*N* = 9950), 56% received *no cancer-directed therapy*, 5% underwent *radiation*, 27% underwent *chemotherapy*, 7% underwent *surgery alone*, and 6% underwent *surgery and chemotherapy*. Compared to *no cancer-directed therapy, radiation therapy* (HR = 0.63), *chemotherapy* (HR = 0.43) *surgery alone* (HR = 0.32), and *surgery and chemotherapy* (HR = 0.23) were all associated with decreased mortality. *Radiation* (AMD = − 3.64), *chemotherapy* (AMD = -6.35), *surgery alone* (AMD = -6.91), and *surgery and chemotherapy* (AMD = -6.74) were all associated with fewer healthcare encounters per 30 days in the last 6 months of life. *Chemotherapy* (AMD = -1.57), *surgery alone* (AMD = -1.65), and *surgery and chemotherapy* (AMD = -1.67) were associated with fewer palliative care visits (all *p*-values for estimates above < 0.05). Treatment groups were associated with lower odds of institutional death and hospitalization at EOL, and higher odds of chemotherapy at EOL.

**Conclusions:**

Receiving cancer-directed therapies was associated with higher survival, fewer healthcare visits, lower odds of dying in an institution and hospitalization at EOL, fewer palliative care visits, and higher odds of receiving chemotherapy at EOL.

**Supplementary Information:**

The online version contains supplementary material available at 10.1186/s12885-022-10342-8.

## Introduction

Pancreatic ductal adenocarcinoma (PDAC) carries a 5-year survival of less than 10% [1, 2] with lower survival among those who are older, more frail, and have more comorbidities [1–4]. Patients with PDAC are faced with challenging treatment decisions immediately following their diagnosis yet often have limited knowledge about the impact of their decisions on their life expectancy, quality of life (QoL), and need for health care services. To inform initial treatment decisions, evidence regarding the association of these decisions with patient-centred outcomes are needed.

Cancer-directed therapy for patients with PDAC includes surgery, chemotherapy, and radiation alone or in combination [5]. Current guidelines emphasize treatment decisions based on cancer location and stage [6, 7]. Patient factors, however, may also impact treatment choices. For instance, by 2030, up to 70% of PDAC cases will be diagnosed in adults over 65 years of age [8], which is a patient demographic historically underrepresented in cancer research [9–11]. Advanced age and associated frailty and comorbidities can impact treatment options due to increased risk of adverse outcomes with therapy [3] and the risk of non-cancer related death [12, 13].

A failure to consider both survival and end-of-life (EOL) outcomes can lead to undertreatment, with the possibility of patients declining treatments due to a low chance of cure and/or meaningful extension in quantity of life. A recent population-based study highlighted that a substantial proportion of patients with PDAC do not access specialized cancer medical or surgical services [14]. Conversely, some patients may be overtreated and exposed to aggressive measures at the end of life aimed at prolonging survival with little efficacy and significant harmful side effects [15]. To address these gaps, we conducted a population-based cohort study to examine the survival and end-of-life (EOL) outcomes among with PDAC patients based on their initial treatment regimen, disease stage, and clinical characteristics.

## Methods

This population-based retrospective cohort study included patients diagnosed with PDAC at any time prior to death in Ontario, Canada. We captured deaths in all Ontarians aged 18 or over between April 1, 2010 and March 31, 2018 and used administrative and clinical databases linked with patient-level identifiers housed at ICES (formerly the Institute of Clinical and Evaluative Sciences). These databases have been used to conduct cohort studies examining end-of-life outcomes for patients with cancer and terminal non-cancer illnesses [16–19]. This study received ethical approval from the Ottawa Health Science Network Research Ethics Board (OHSN-REB). Informed consent was waived due to the retrospective nature of the study by the OHSN-REB.

All methods were carried out in accordance with Declaration of Helsinki. We reported this study according to the Reporting of studies Conducted using Observational Routinely-collected health Data (RECORD) statement [20].

### Data Sources

We identified patients with PDAC through the Ontario Cancer Registry (OCR), which contains information on incident cancers diagnosed since 1964 and is approximately 95% complete [21, 22], and used encrypted provincial health insurance numbers to link databases via ICES (Supplementary Table 1). We used the Registered Persons Database [23] and Vital Statistics registry [24] to obtain demographic and vital status data respectively. To obtain information about health services utilization, we used the Ontario Health Insurance Plan (OHIP) claims database [25] (healthcare provider billings), the Canadian Institute for Health Information (CIHI) Discharge Abstract Database (DAD) [26] and Same Day Surgery Database (SDS) [27], Cancer Activity Level Reporting (ALR) database (radiation, chemotherapy, and oncology appointment visits) [28], National Ambulatory Care Reporting System [29], National Rehabilitation Reporting System (NRS) [30], and Continuing Care Reporting System [31]. We obtained information on homecare through the InterRAI Reporting System [32, 33] (homecare assessments) and the Home Care Database (HCD) [34] (services provided or coordinated by local health networks in Ontario). All database codes used in this study are available in Supplementary Table 2.

### Cohort

We included patients who died from April 1, 2010 to December 31, 2017 and had a diagnosis of PDAC prior to death based on ICD-10 (International Statistical Classification of Diseases and Related Health Problems 10th revision) codes [35] (Supplementary Table 2). We excluded patients who were not eligible for Ontario health insurance coverage at their PDAC diagnosis date, or who lost eligibility before their death or the end of the study period. These patients would not have been captured in administrative databases, which are linked through health insurance numbers. We also excluded patients aged under 18 years or greater than 105 years, whose age was not known, and those diagnosed with possible neuroendocrine tumors.

### Index cancer treatment

The primary exposure was index cancer treatment, with five a priori defined treatment groups: *no cancer-directed therapy, radiation, chemotherapy, surgery only, surgery and chemotherapy.*

The *surgery and chemotherapy* group was defined as having undergone surgical intervention for pancreatic cancer with pancreatectomy and the receipt of at least one dose of chemotherapy within 120 days of index cancer diagnosis. The *surgery only* group was defined as having undergone surgical intervention for pancreatic cancer with pancreatectomy but no chemotherapy in the initial 120 days after diagnosis. The *chemotherapy* group was defined as having received at least one dose of chemotherapy, with no pancreatectomy, within 120 days of diagnosis. This included patients who underwent initial chemoradiation treatment. The *radiation* group was defined as having received at least one dose of radiation therapy with no pancreatectomy or chemotherapy in the initial 120 days after diagnosis. The *no cancer-directed therapy* group was defined as having received none of radiation, chemotherapy, or pancreatectomy within 120 days of diagnosis. Surgery, radiation, and chemotherapy treatments were identified using OHIP claims and ALR codes (Supplementary Table 2) [14, 36–38]. As we were interested in index treatment decisions, we did not group patients based on treatments they received after 120 days from diagnosis.

### Covariates

We collected data on age, sex, rural location of residence, neighborhood income quintile, comorbidities, location of primary pancreatic tumor, cancer stage, and index cancer treatment. We used the Charlson Comorbidity Index (CCI) to characterize comorbidities [39], with patients categorized as having a CCI score of 0, 1, or ≥ 2. Comorbidities included 18 chronic conditions (acute myocardial infarction, arrhythmia, asthma, cancer, congestive heart failure, chronic obstructive pulmonary disease, coronary artery disease, dementia, diabetes, hypertension, inflammatory bowel disease, non-psychotic mood and anxiety disorders, other mental health illnesses, osteoarthritis, osteoporosis, renal disease, rheumatoid arthritis, and stroke) with high prevalence and economic burden in Ontario [40–42].

OCR data were used for tumor location and cancer staging [43–45] to derive the “best stage” grouping consistent with the American Joint Committee on Cancer staging manual [46]. Residence was defined as rural (community size greater than 10,000 persons) or urban using postal codes in the Registered Persons Database (RPDB), and income quintile as the median income of a patient’s postal code using Canadian census data [47, 48]. Cohort identification, demographic variables, and covariate codes are available in Supplementary Table 3.

### Outcomes

The primary outcomes were survival, healthcare encounters per 30 days in the last 6 months of life, and palliative care visits per 30 days within the last 6 months of life. We defined healthcare encounters as primary care visits, emergency department visits, and hospital admissions (including intensive care admissions). Palliative care visits included outpatient visits, homecare visits, and palliative inpatient admissions [19]. We captured inpatient and outpatient palliative care treatments based on a set of CIHI DAD, HCD, and OHIP claims codes [49–51]. Secondary outcomes included location of death, hospitalization within the last 30 days of life, and receipt of chemotherapy within the last 30 days of life. We categorized location of death as institutional (emergency department, acute care ward, intensive care unit, complex continuing care and rehabilitation facility, long-term care) and community (home or hospice) [52]. Hospitalization and chemotherapy within the last the last 30 days of life were selected as measures of aggressive EOL care, in keeping with prior studies [53–55]. All database codes for outcomes are available in Supplementary Table 4.

### Data analysis

We calculated descriptive statistics for all patient at the time of their diagnosis. Variables and outcomes were stratified by index cancer treatment. We estimated the association between the exposure variables and outcomes using multivariable models. Survival was modelled with Cox regression, rates of end-of-life healthcare encounters and palliative care visits per 30 days were modelled using linear regression, and binary outcomes (location of death as institution vs. community, any hospitalization within the last 30 days of life, and any receipt of chemotherapy within the last 30 days of life) were modelled using logistic regression.

We adjusted all models for age, sex, rurality, neighbourhood income, comorbidity, location of the tumor in the pancreas and cancer stage. We performed one sensitivity analysis planned a priori by excluding patients without cancer stage data because we theorized that these patients were less likely to have undergone cancer-directed therapy. Hazard ratio, adjusted mean difference, and odds ratios were reported for Cox regression, linear regression, and logistic regression respectively, all with 95% confidence intervals. A *P*-value of < 0.05 was considered statistically significant for all analyses.

## Results

For the study period, 9950 adults had a diagnosis of PDAC captured in OCR prior to their death. The median age at diagnosis was 78 years (IQR 64–812%), 51% were women, and 86% lived in an urban location. The Charlson comorbidity index was 0 for 84%, 1 for 6%, and greater than 1 for 9% of patients. At diagnosis, 2% of patients had stage 1 disease, 8% stage 2, 7% stage 3, 26% stage 4, and 57% were missing stage data (Table 1). With respect to initial treatments, 56% received no cancer-directed therapy, 5% underwent radiation, 27% underwent chemotherapy, 7% underwent surgery alone, and 6% underwent surgery and chemotherapy.

**Table 1:** PDAC cohort demographics and baseline data by initial treatment group

	No cancer-directed therapy	Radiation	Chemotherapy	Surgery alone	Surgery and chemotherapy	Total
	*N* = 5551	*N* = 461	*N* = 2677	*N* = 692	*N* = 569	*N* = 9950
Age at diagnosis, median (IQR)	78 (68–84)	73 (64–81)	66 (59–73)	70 (62–76)	66 (59–72)	72 (64–81)
Female sex, n (%)	2831 (51%)	226 (49%)	1175 (44%)	307 (44%)	264 (46%)	4803 (48%)
Income quintile, n (%)						
1st (lowest)	1296 (23%)	100 (22%)	442 (17%)	139 (20%)	87 (15%)	2064 (21%)
2nd	1156 (21%)	95 (21%)	537 (20%)	138 (20%)	119 (21%)	2045 (21%)
3rd	1137 (20%)	94 (20%)	518 (19%)	133 (19%)	109 (19%)	1991 (20%)
4th	1033 (19%)	101 (22%)	566 (21%)	136 (20%)	106 (19%)	1942 (19%)
5th (highest)	920 (17%)	71 (15%)	611 (23%)	145 (21%)	148 (26%)	1895 (19%)
Rural place of residence, n (%)	772 (13%)	70 (15%)	316 (12%)	114 (17%)	81 (14%)	1353 (14%)
Comorbidities, n (%)						
CHF	874 (16%)	34 (7%)	95 (4%)	35 (5%)	17 (3%)	1055 (11%)
COPD	806 (14%)	65 (14%)	193 (7%)	61 (9%)	35 (6%)	1160 (12%)
CAD	1795 (32%)	135 (29%)	504 (19%)	167 (24%)	88 (16%)	2689 (27%)
Stroke	493 (9%)	27 (6%)	80 (3%)	27 (4%)	14 (3%)	641 (6%)
Renal disease	604 (11%)	39 (9%)	93 (4%)	37 (5%)	18 (3%)	791 (8%)
Charlson comorbidity index, n (%)						
0	4458 (80%)	395 (86%)	2420 (90%)	595 (86%)	515 (90%)	8383 (84%)
1	393 (7%)	25 (5%)	124 (5%)	53 (8%)	32 (6%)	627 (6%)
≥ 2	700 (13%)	41 (9%)	133 (5%)	44 (6%)	22 (4%)	940 (9%)
Disease stage at diagnosis, n (%)						
1	86 (1%)	20 (4%)	28 (1%)	19 (3%)	14 (2%)	167 (2%)
2	95 (2%)	25 (5%)	112 (4%)	294 (42%)	306 (54%)	832 (8%)
3	199 (4%)	74 (16%)	392 (15%)	36 (5%)	31 (5%)	732 (7%)
4	1099 (20%)	180 (39%)	1169 (44%)	66 (10%)	55 (10%)	2569 (26%)
Missing stage	4072 (73%)	162 (35%)	976 (36%)	277 (40%)	163 (29%)	5650 (57%)
Tumor location within pancreas, n (%)						
Head	1961 (35%)	198 (43%)	1176 (44%)	514 (74%)	427 (75%)	4276 (43%)
Body	473 (9%)	71 (15%)	450 (17%)	56 (8%)	44 (8%)	1094 (11%)
Tail	592 (11%)	65 (14%)	424 (16%)	70 (10%)	68 (12%)	1219 (12%)
Duct	39 (1%)	5 (1%)	27 (1%)	9 (1%)	6 (1%)	84 (1%)
Neck	292 (5%)	37 (8%)	195 (7%)	16 (2%)	13 (2%)	553 (6%)
Unspecified	2194 (39%)	87 (19%)	405 (15%)	27 (4%)	11 (2%)	2724 (27%)

*IQR* interquartile range, *CHF* congestive heart failure, *COPD* chronic obstructive pulmonary disease, *CAD* coronary artery disease

### Survival, healthcare encounters, and palliative care

**Fig. 1:**
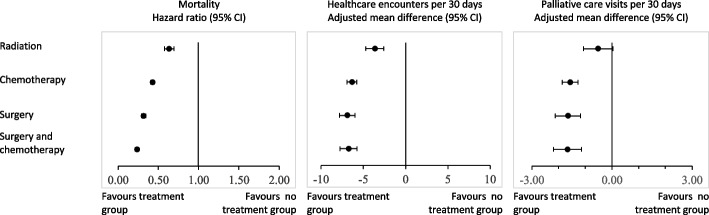
Association between index cancer treatment and primary outcomes

Median patient survival was 105 days (IQR 37–227) (Table 2). In the multivariable Cox regression, *radiation therapy* (hazard ratio [HR] = 0.63, 95% confidence interval [CI] = 0.57 to 0.70), *chemotherapy* (HR = 0.43, 95% CI = 0.41 to 0.45) *surgery alone* (HR = 0.32, 95% CI = 0.29 to 0.34), and *surgery and chemotherapy* (HR = 0.23, 95% CI = 0.21 to 0.26) were all associated with decreased mortality, compared to *no cancer-directed therapy* (Table 3, Fig. 1).

**Table 2:** Unadjusted estimates of each outcome for entire cohort by initial treatment group

	No cancer-directed therapy	Radiation	Chemotherapy	Surgery alone	Surgery and chemotherapy	Total
	*N* = 5551	*N* = 461	*N* = 2677	*N* = 692	*N* = 569	*N* = 9950
Survival, median days from diagnosis (IQR)	47 (22–107)	120 (64–218)	224 (122–373)	326 (158–559)	494 (304–746)	105 (37–277)
Healthcare encounters per 30 days, median (IQR)	9 (5–18)	7 (4–13)	5 (3–8)	4 (2–7)	4 (3–7)	7 (4–12)
Palliative care visits per 30 days, median (IQR)	1 (0–6)	1 (0–6)	0 (0–4)	0 (0–3)	0 (0–4)	1 (0–5)
Institutional death^a^, n (%)	3557 (64%)	263 (57%)	1425 (53%)	400 (56%)	295 (52%)	5940 (60%)
Hospitalization within last 30 days of life, n (%)	3347 (60%)	234 (51%)	1457 (54%)	342 (49%)	285 (50%)	5665 (57%)
Chemotherapy within last 30 days of life, n (%)	33 (1%)	11 (2%)	722 (27%)	41 (6%)	68 (12%)	875 (9%)

*IQR* interquartile range, *SD* standard deviation

^a^Includes emergency department, acute care ward, intensive care unit, complex continuing care and rehabilitation facility, long-term care

**Table 3:** Multivariate^a^ adjusted estimates for each outcome by initial treatment group

	Mortality	Healthcare encounters per 30 days	Palliative care visits per 30 days	Institutional death^b^	Hospitalization within last 30 days of life	Chemotherapy within last 30 days of life
	HR (95% CI)	AMD (95% CI)	AMD (95% CI)	OR (95% CI)	OR (95% CI)	OR (95% CI)
**Initial treatment**						
No cancer-directed therapy	Referent	Referent	Referent	Referent	Referent	Referent
Radiation	0.63 (0.57 to 0.70)	-3.64 (−4.71 to −2.58)	−0.52 (−1.07 to 0.03)	0.79 (0.66 to 0.97)	0.66 (0.54 to 0.80)	4.13 (2.06 to 8.27)
Chemotherapy	0.43 (0.41 to 0.45)	−6.35 (−6.92 to −5.78)	− 1.57 (− 1.87 to − 1.28)	0.72 (0.65 to 0.79)	0.72 (0.65 to 0.80)	57.44 (39.71 to 83.08)
Surgery alone	0.32 (0.29 to 0.34)	−6.91 (−7.82 to −6.00)	−1.65 (−2.13 to − 1.18)	0.86 (0.73 to 1.02)	0.63 (0.53 to 0.74)	10.77 (6.66 to 17.43)
Surgery and chemotherapy	0.23 (0.21 to 0.26)	−6.74 (−7.75 to −5.74)	−1.67 (−2.19 to − 1.14)	0.70 (0.59 to 0.85)	0.63 (0.52 to 0.75)	21.81 (13.94 to 34.12)
Age	1.01 (1.01 to 1.01)	0.05 (0.03 to 0.07)	0.02 (0.01 to 0.03)	1.00 (1.00 to 1.00)	0.99 (0.98 to 0.99)	0.99 (0.98 to 0.99)
Female sex	0.93 (0.88 to 0.99)	0.09 (−0.35 to 0.53)	0.24 (0.02 to 0.47)	0.95 (0.88 to 1.03)	0.77 (0.71 to 0.83)	0.79 (0.67 to 0.92)
Rural place of residence	1.13 (1.04 to 1.23)	2.34 (1.70 to 3.00)	−0.47 (− 0.80 to − 0.13)	0.93 (0.82 to 1.04)	1.15 (1.02 to 1.30)	0.95 (0.75 to 1.20)
Lowest quintile	Referent	Referent	Referent	Referent	Referent	Referent
Highest quintile	0.99 (0.90 to 1.09)	−0.20 (−0.90 to 0.50)	0.94 (0.58 to 1.30)	0.64 (0.56 to 0.73)	0.78 (0.68 to 0.88)	1.31 (1.01 to 1.69)
Charlson comorbidity index	1.05 (1.01 to 1.09)	0.63 (0.43 to 0.83)	0.01 (−0.10 to 0.11)	1.09 (1.05 to 1.14)	1.03 (0.99 to 1.07)	0.98 (0.90 to 1.07)

*HR* hazard ratio, *AMD* absolute mean difference, *CI* confidence interval, *OR* odds ratio

^a^Cox regression used to model survival, linear regression used to model rates of healthcare encounters and palliative care visits per 30 days, and logistic regression to model institutional death, hospitalization within 30 days of life, and chemotherapy within 30 days of life

^b^Includes emergency department, acute care ward, intensive care unit, complex continuing care and rehabilitation facility, long-term care

Patients had a median of 7 (IQR 3–12) healthcare encounters per 30 days within the last 6 months of life, ranging from 4 (IQR 2–7) days in the *surgery alone* group to 9 (IQR 5–18) in the *no cancer-directed therapy* group (Table 2). Compared to *no cancer-directed therapy*, *radiation* (adjusted mean difference [AMD] = − 3.64, 95% CI = − 4.71 to − 2.58), *chemotherapy* (AMD = − 6.35, 95% CI = − 6.92 to − 5.78), *surgery alone* (AMD = − 6.91, 95% CI = − 7.82 to − 6.00), and *surgery and chemotherapy* (AMD = − 6.74, 95% CI = − 7.75 to − 5.74) were all associated with fewer healthcare encounters per 30 days in the last 6 months of life Table 3, Fig. 1).

Patients had a median of 1 (IQR 0–5) palliative care visits per 30 days within the last 6 months of life, ranging from 0 (IQR 0–3) days in the *surgery alone* group to 1 (IQR 0–6) in the *no cancer-directed therapy* and *radiation* groups (Table 2). *Chemotherapy* (AMD = − 1.57, 95% CI − 1.87 to − 1.28), *surgery alone* (AMD = − 1.65, 95% CI − 2.13 to − 1.18), and *surgery and chemotherapy* (AMD = − 1.67, 95% CI − 2.19 to − 1.14) were associated with fewer palliative care visits compared to *no cancer-directed therapy*, while *radiation* treatment was not Table 3, Fig. 1).

### Location of death and interventions near the end of life

A total of 60% of patients died in an institution, 57% were hospitalized in the last 30 days of life, and 9% received chemotherapy in the last 30 days of life. *Radiation, chemotherapy*, and *surgery and chemotherapy* were associated with lower odds of institutional death compared to *no cancer-directed therapy*. All treatment groups had lower odds of hospitalization within the last 30 days of life and higher odds of chemotherapy within the last 30 days of life compared to the *no cancer-directed therapy* group (Table 3).

### Sensitivity analysis

Trends with respect to primary outcomes did not change in the sensitivity analysis, except for *radiation* group. After excluding cases with no information regarding cancer stage, 4300 (43% of all included patients) were considered in the multivariable analyses (Table 4). All treatment groups except the radiation group had lower mortality, and lower adjusted mean healthcare encounters and palliative care visits within the last 6 months of life compared to the *no cancer-directed therapy* group.

**Table 4:** Sensitivity analysis of multivariable regression analyses excluding patients without stage data ^a^

	Mortality	Healthcare encounters per 30 days	Palliative care visits per 30 days	Institutional death^b^	Hospitalization within last 30 days of life	Chemotherapy within last 30 days of life
	HR (95% CI)	AMD (95% CI)	AMD (95% CI)	OR (95% CI)	OR (95% CI)	OR (95% CI)
No cancer-directed therapy (*N* = 1479)	Referent	Referent	Referent	Referent	Referent	Referent
Radiation (*N* = 299)	0.97 (0.85 to 1.10)	−0.09 (−0.76 to 0.57)	−0.47 (−1.11 to 0.17	0.91 (0.71 to 1.17)	0.92 (0.72 to 1.19)	2.24 (1.02 to 4.92)
Chemotherapy (*N* = 1701)	0.49 (0.45 to 0.53)	−2.96 (−3.35 to −2.56)	−1.50 (− 1.87 to − 1.12)	0.93 (0.80 to 1.08)	0.92 (0.79 to 1.06)	21.66 (13.94 to 33.67)
Surgery alone (*N* = 415)	0.42 (0.37 to 0.48)	−2.76 (−3.45 to − 2.07)	−1.23 (− 1.88 to − 0.57)	0.83 (0.64 to 1.07)	0.74 (0.57 to 0.96)	7.86 (4.30 to 14.36)
Surgery and chemotherapy (*N* = 406)	0.37 (0.32 to 0.42)	−2.55 (− 3.27 to − 1.84)	−1.01 (− 1.69 to − 0.33)	0.70 (0.54 to 0.92)	0.77 (0.59 to 1.01)	13.51 (7.62 to 23.97)

*HR* hazard ratio, *AMD* absolute mean difference, *CI* confidence interval, *OR* odds ratio

^a^Cox regression used to model survival, linear regression used to model healthcare encounters and palliative care visits per 30 days, and logistic regression to model institutional death, hospitalization within 30 days of life, and chemotherapy within 30 days of life

^b^Includes emergency department, acute care ward, intensive care unit, complex continuing care and rehabilitation facility, long-term care

With respect to secondary outcomes, only the *surgery and chemotherapy* group had lower odds of institutional death compared to *no cancer-directed therapy*, and only the *surgery alone* group had lower odds of hospitalization within the last 30 days of life compared to *no cancer-directed therapy*. All treatment groups had higher odds of receiving chemotherapy within 30 days of death compared to the *no cancer-directed therapy group*.

## Discussion

In this population-based study examining the relationship between initial treatment and the end-of-life experience of patients with PDAC in Ontario, we found that patients who received cancer-directed therapy tended to have lower mortality, fewer healthcare encounters, lower odds of hospitalization at the end of life and lower odds of institutional death compared to patients with no cancer-directed therapy. Patients who did not receive initial treatment had more palliative care visits and lower odds of receiving chemotherapy at the end of life. Similar trends were observed after excluding patients without cancer stage data. Our study offers patients, their caregivers, and healthcare providers clinically relevant information regarding associations between initial cancer-directed treatments and end-of-life experiences.

Our finding of higher healthcare encounters among patients with no initial cancer-directed treatment suggests that these patients may have fewer outpatient resources and/or higher symptom burden towards the end of their life. While patients may forego cancer-directed therapies due to knowledge about poor prognosis, lack of treatment may also be due to low rates of specialist consultation and subsequent informed discussions regarding such therapies [14]. Despite initial negative views on the benefits and risks associated with chemotherapy, many patients go on receive chemotherapy after engaging in shared-decision making with their oncologists [56]. Without specialist consultation, patients may not gain access to cancer-directed therapy that can impact survival [57, 58] or palliative surgical options to improve QoL such as gastrojejunostomy for gastric outlet obstruction [59]. This finding of higher healthcare utilization for these patients may highlight a gap in their care.

By contrast, patients who did receive cancer-directed therapy in our study were almost certainly connected with oncology specialists, as chemotherapy, radiation, and pancreatectomy are provided by medical oncologists, radiation oncologists, and hepatobiliary surgeons respectively in Ontario [36, 38, 60, 61]. These patients, however, tended to have fewer outpatient, home, and inpatient palliative care visits. They were also more likely to have received chemotherapy within 30 days of death. Systemic chemotherapy, despite a potential role symptom relief, is associated with poor QoL at the end of life [62]. These findings are consistent with prior literature showing that palliative care involvement is associated with less aggressive care near death [63, 64]. Though lower palliative care utilization may be due to better symptom control, the increased use of chemotherapy in patients who received initial cancer-directed treatment may indicates that certain patients and their providers continue to pursue aggressive treatments near death [65]. Patient awareness of this trend and informed discussions on the benefits of palliative care can support treatment decisions.

This study has several limitations. First, stage data was missing for many included patients. While this did not affect the majority of trends identified in our study based on sensitivity analyses, we note that the *radiation* group did not have sifnicantly different primary outcomes compared to no treatment when excluding stage data. Second, administrative databases are not complete and lack details on elements such as patient performance status. Use of these databases may also lead to case ascertainment bias, as not all patients had pathologic confirmation of the diagnosis. Third, as administrative data sets are not collected to address specific research questions, we lacked patient- and provider-specific details on preferences for care. Finally, we did not account for regional variations in care or for the effect of institutional patient volume on clinical outcomes [66], as this was beyond the scope of this study.

This study also has several key strengths. Our use of high-quality population level databases adds generalizability to our findings. Additionally, we utilized clinically relevant variables, and characterized demographic, clinical, and outcomes data using previously validated codes through administrative databases. While prior studies on end-of-life outcomes in this field have focused on the role of palliative care, aggressive interventions near death, and healthcare resource utilization [16, 62, 63, 67], our findings are unique as they provide estimates based on index cancer treatment.

## Conclusions

Patients who received cancer-directed therapy for PDAC had greater survival, fewer healthcare encounters, lower odds of hospitalization at the end of life and lower odds of institutional death compared to patients with no cancer-directed therapy. Patients who did not receive initial treatment had more palliative care visits and lower odds of receiving chemotherapy at the end of life. These data can inform initial treatment decisions for patients after a diagnosis of PDAC.

## Supplementary Information

**Additional file 1.**

## Data Availability

All data generated or analysed during this study are included in this published article [and its supplementary information files].
