# Supplementary material for: Initial treatment is associated with improved survival and end-of-life outcomes for patients with pancreatic cancer: a cohort study
Source: BMC Cancer. 2022 Dec 14;22:1312. doi: 10.1186/s12885-022-10342-8 (PMC9753384; doi:10.1186/s12885-022-10342-8)
Supplement: Supplementary file 1 — Additional file 1. [file 12885_2022_10342_MOESM1_ESM.doc]

**Supplementary table 1:** Administrative databases linked through ICES

| **Database** | **Description** |
| --- | --- |
| Canadian Institute for Health Information (CIHI) Discharge Abstract Database (DAD)^1^ and Same Day Surgery (SDS) Database^2^ | Discharges from acute care facilities and same day surgeries |
| Cancer Activity Level Reporting (ALR)^3^ | Patient level activity within the cancer system focused on radiation, chemotherapy, and outpatient oncology clinic visits |
| Continuing Care Reporting System^4^ | Resource utilization by individuals receiving continuing care services in hospitals or long-term care homes |
| Home Care Database (HCD)^5^ | Homecare services provided or coordinated by Ontario Local Health Integration Networks |
| InterRAI Reporting System^6,7^ | Homecare assessment data |
| Ontario Cancer Registry (OCR) ^8^ | Cancer diagnoses, morphology, and staging |
| Ontario Health Insurance Plan (OHIP) claims database^9^ | Physician services billing data for services provided to Ontario’s residents |
| National Ambulatory Care Reporting System (NACRS)^10^ | Emergency department visit data |
| National Rehabilitation Reporting System (NRS)^11^ | Clinical data on functional status (cognitive function and the performance of instrumental activities of daily living) from participating adult inpatient rehabilitation facilities and programs across Canada. |
| Registered Persons Database (RPDB)^12^ | Sociodemographic information |
| Vital Statistics Registry^13^ | Date and cause of death |

**Supplementary table 2:** Index treatment identified using Ontario healthcare administrative databases.

| **Variable** | **Data source** | **Codes** |
| --- | --- | --- |
| Radiation therapy | ALR | Radiation treatment codes  CODE: 120, 130, 503, 510, 511, 512, 519, 520, 521, 522, 523, 524, 525, 526, 527, 528, 529, 530, 531, 532, 533, 534, 535, 536, 537, 538, 539, 540, 541, 542, 548, 549, 561, 563, 565, 566, 568, 570, 571, 572, 573, 574, 575, 581, 582, 590, 591, 592, 594, 596, 597  Treatment date: “Ready_to_treat_date” = not missing, AND “dose_per_fraction” not 0 AND >0 |
| Chemotherapy | OHIP | Chemotherapy treatment  OHIP FEECODE: G281, G339, G345, G359, G381 |
| Pancreatectomy | OHIP | S298 Pancreatectomy – Complete with splenectomy  S299 Pancreatectomy – Distal body, tail with preservation of spleen, with or without anastomosis  S300 Pancreatectomy – “Whipple type” procedure  S301 Pancreatectomy – Local complete excision of tumor or lesion  S309 Pancreatectomy – Distal body, tail with splenectomy with or without anastomosis |

**Supplementary table 3:** Cohort identification, demographic, and covariates identified using Ontario healthcare administrative databases.

| **Variable** | **Data source** | **Codes** |
| --- | --- | --- |
| Pancreatic adenocarcinoma diagnosis | OCR | International Classification of Diseases (ICD) diagnostic codes  ICD-O-3 DXCODE: C25.0-C25.3,C25.5-C25.9  Morphology ([CURR_MORPH_CD](javascript:__doPostBack('ctl00$MainContent$gvList$ctl74$VariableLink',''))): 8000, 8001, 8010, 8020, 8021, 8031, 8035, 8140, 8144, 8145, 8255, 8340, 8341, 8344, 8440, 8442, 8470, 8481, 8490, 8500, 8560, 8570, 8574, 8575, 9990  Note: excluded neuroendocrine carcinomas: ICD-O-3 C25.4 |
| Cancer staging | OCR | “Best stage” grouping consistent with the American Joint Committee on Cancer staging manual^14^  BEST_STAGE_GRP  Stage 0 - 000  Stage I - 100  Stage IA - 120  Stage IB - 150  Stage II - 300  Stage IIA - 320  Stage IIB - 330  Stage III – 500  Stage IV - 700  Missing stage– no code |
| Tumor location | OCR | ICD diagnostic codes (ICD-O-3: DXCODE)  Head of pancreas – C25.0  Neck of pancreas – C25.7 or C25.8  Duct of pancreas – C25.3  Body of pancreas – C25.1  Tail of pancreas – C25.2  Unspecified – C25.9 |
| Age and sex | RPDB | Male or female using variable SEX  Age at diagnosis using variable AGEDX |
| Rural residence | RPDB | Rural status (community size <10,000) calculated based on postal code (PSTLCODE) |
| Income quintile | RPDB | Geographic income estimation^15^ using variable INQUINT, which estimates income quintile as the median income of a patient’s postal code using Canadian census data^15,16^. |
| Comorbidities | CIHI DAD | Operationalized with Charlson comorbidity index^17^ (categorized as score of 0, 1, ≥2), and includes 18 chronic conditions (acute myocardial infarction, arrhythmia, asthma, cancer, congestive heart failure, chronic obstructive pulmonary disease, coronary artery disease, dementia, diabetes, hypertension, inflammatory bowel disease, non-psychotic mood and anxiety disorders, other mental health illnesses, osteoarthritis, osteoporosis, renal disease, rheumatoid arthritis, and stroke) with high prevalence and economic burden in Ontario^18–20^. |

**Supplementary table 4:** Outcome variables identified using Ontario healthcare administrative databases.

| **Variable** | **Data source** | **Codes** |
| --- | --- | --- |
| Mortality (from date of diagnosis) | OCR  RPDB | Date of death using variable DTHDATE (RPDB), estimated from pancreatic cancer diagnosis date with variable pcdiag_date (OCR) |
| Healthcare encounters | CIHI DAD  NACRS | Primary care visits: number of encounters with a primary care physician using FEECODES (Spec = 00, 05 OR mainspecialty = GP/FP or community medicine)  Emergency department visits: any ED visit record with source variable E  Hospitalizations: number of hospitalizations using variable [ADMDATE](javascript:__doPostBack('ctl00$MainContent$gvList$ctl10$VariableLink','')) |
| Palliative care utilization | CIHI DAD HCD  OHIP | Palliative inpatient services identified:  CIHI DAD codes (DX10CODE[1-25]=Z515  or if PATSERV=58)  OHIP (while patient is in hospital): FEECODE = C945, C882, C982, A945, K015, K023  Palliative homecare services:  HCD Service Recipient Code 95  OHIP: FEECODE = A900-901, B960-964, B966, B986-988, B990, B992-994, B996-998 |
| Location of death | CCRS  CIHI DAD  CIHI SDS  HCD  OHIP  NACRS  NRS | Categorized as institution versus community.  Institution:  Acute care (emergency department, acute care ward, intensive care unit) – DAD, NACRS, SDS  Sub-acute (complex continuing care and rehabilitation facility) - CCRS-CCC, NRS, OMHRS  Long-term care - CCRS-LTC  Community:  home or hospice - HCD, OHIP |
| Hospitalization within last 30 days of life | CIHI DAD | Any hospitalizations using variable [ADMDATE](javascript:__doPostBack('ctl00$MainContent$gvList$ctl10$VariableLink','')) |
| Chemotherapy within last 30 days of life | OHIP | Any chemotherapy treatment  OHIP FEECODE: G281, G339, G345, G359, G381 |

**References**

1. Canadian Institute for Health Information. Discharge Abstract Data metadata. cihi.ca. Accessed November 25, 2020. https://www.cihi.ca/en/discharge-abstract-database-metadata-dad

2. ICES. Data Dictionary: SDS. datadictionary.ices.on.ca. Published 2022. Accessed April 2, 2022. https://datadictionary.ices.on.ca/Applications/DataDictionary/Library.aspx?Library=SDS

3. Cancer Care Ontario’s Data Book - 2018-2019. I. Cancer Activity Level Reporting (ALR). cancercareontario.ca. Accessed March 31, 2022. https://ext.cancercare.on.ca/ext/databook/db1819/I-_Activity_Level_Reporting_ALR/Introduction.htm

4. Canadian Institute for Health Information. Continuing Care Reporting System. cihi.ca. Accessed December 13, 2020. https://www.cihi.ca/en/continuing-care

5. Health Quality Ontario. Measuring Home Care Performance in Ontario. hqontario.ca. Accessed December 13, 2020. https://hqontario.ca/System-Performance/Measuring-System-Performance/Measuring-Home-Care

6. The InterRAI Organization. InterRAI. interrai.org. Accessed December 13, 2020. https://www.interrai.org/

7. Canadian Institute for Health Information. Home Care. cihi.ca. Accessed December 13, 2020. https://www.cihi.ca/en/home-care

8. Clarke E, Marrett L, Kreiger N. Cancer registration in Ontario: a computer approach. *IARC Sci Publ*. 1991;95:246-257.

9. Government of Ontario. Health Care in Ontario. Ontario.ca. Accessed November 25, 2020. https://www.ontario.ca/page/health-care-ontario

10. Canadian Institute for Health Information. National Ambulatory Care Reporting System metadata (NACRS). cihi.ca. Accessed November 25, 2020. https://www.cihi.ca/en/national-ambulatory-care-reporting-system-metadata-nacrs

11. Canadian Institute for Health Information. National Rehabiliation Reporting System. cihi.ca. Published 2021. Accessed April 4, 2022. https://www.cihi.ca/en/national-rehabilitation-reporting-system-metadata

12. Government of Ontario. Registered Persons Database. data.ontario.ca. Accessed November 25, 2020. https://data.ontario.ca/dataset/registered-persons-database-rpdb

13. Library and Archives Canada. Births, Marriages, and Deaths Recorded in Canada. bac-lac.gc.ca. Accessed November 25, 2020. https://www.bac-lac.gc.ca/eng/discover/vital-statistics-births-marriages-deaths/births-marriages-deaths-recorded/Pages/births-marriages-deaths-recorded.aspx#b

14. Edge S, Compton C, Fritz A, Greene F, Trotti A, (Editors). *American Joint Committee on Cancer. AJCC Cancer Staging Manual.* 7th Edition. Springer; 2010.

15. Alter DA, Naylor CD, Austin P, Tu JV. Effects of socioeconomic status on access to invasive cardiac procedures and on mortality after acute myocardial infarction. *New England Journal of Medicine*. 1999;341(18):1359-1367.

16. Wilkins R. Use of postal codes and addresses in the analysis of health data. *Health Reports*. 1993;5(2):157-177.

17. Charlson M, Szatrowski TP, Peterson J, Gold J. Validation of a combined comorbidity index. *Journal of clinical epidemiology*. 1994;47(11):1245-1251.

18. Pefoyo AJK, Bronskill SE, Gruneir A, et al. The increasing burden and complexity of multimorbidity. *BMC public health*. 2015;15(1):1-11.

19. Thavorn K, Maxwell CJ, Gruneir A, et al. Effect of socio-demographic factors on the association between multimorbidity and healthcare costs: a population-based, retrospective cohort study. *BMJ open*. 2017;7(10):e017264.

20. Kone AP, Mondor L, Maxwell C, Kabir US, Rosella LC, Wodchis WP. Rising burden of multimorbidity and related socio-demographic factors: a repeated cross-sectional study of Ontarians. *Canadian Journal of Public Health*. 2021;112(4):737-747.
